# CRISPR/Cas9 Mediated Genome Engineering for Improvement of Horticultural Crops

**DOI:** 10.3389/fpls.2017.01635

**Published:** 2017-09-22

**Authors:** Suhas G. Karkute, Achuit K. Singh, Om P. Gupta, Prabhakar M. Singh, Bijendra Singh

**Affiliations:** ^1^Division of Vegetable Improvement, ICAR-Indian Institute of Vegetable Research Varanasi, India; ^2^Division of Quality and Basic Sciences, ICAR-Indian Institute of Wheat and Barley Research Karnal, India

**Keywords:** CRISPR/Cas9, genome editing, vegetables, fruits, horticultural crops

## Abstract

Horticultural crops are an important part of agriculture for food as well as nutritional security. However, several pests and diseases along with adverse abiotic environmental factors pose a severe threat to these crops by affecting their quality and productivity. This warrants the effective and accelerated breeding programs by utilizing innovative biotechnological tools that can tackle aforementioned issues. The recent technique of genome editing by Clustered Regularly Interspaced Short Palindromic Repeats/CRISPR associated 9 (CRISPR/Cas9) has greatly advanced the breeding for crop improvement due to its simplicity and high efficiency over other nucleases such as Zinc Finger Nucleases and Transcription Activator Like Effector Nucleases. CRISPR/Cas9 tool contains a non-specific Cas9 nuclease and a single guide RNA that directs Cas9 to the specific genomic location creating double-strand breaks and subsequent repair process creates insertion or deletion mutations. This is currently the widely adopted tool for reverse genetics, and crop improvement in large number of agricultural crops. The use of CRISPR/Cas9 in horticultural crops is limited to few crops due to lack of availability of regeneration protocols and sufficient sequence information in many horticultural crops. In this review, the present status of applicability of CRISPR/Cas9 in horticultural crops was discussed along with the challenges and future potential for possible improvement of these crops for their yield, quality, and resistance to biotic and abiotic stress.

## Introduction

Horticulture is an important and integral part of the economy that contributes significantly to the total agricultural production. The horticulture covers several crops like vegetables, fruits, flowers, tuber crops, spices, medicinal and aromatic plants, and plantation crops. Among all, fruits and vegetables have huge yield potential. Besides, they are rich source of nutrients that are beneficial to human health (Slavin and Lloyd, 2012) and thus play an important role in providing food and nutritional security. To ensure sufficient food supply to ever-increasing human population across the globe, we must ensure accelerated growth in crop production. Vegetable and fruit crops are more prone to the damages by climate change necessitating the development of next generation crops that can sustain in adverse climatic conditions. Conventional breeding for crop improvement has been extensively used despite the fact that it is a time-consuming practice (Ashraf, 2010; Tester and Langridge, 2010). Its contribution to crop improvement is limited by declining genetic base that further depends on existing natural allelic variations and crossing barriers in cultivated and wild species. Transgenesis is the potential alternative to plant breeding to overcome the crossing barrier but public acceptance is a serious concern that prevents commercialization of transgenic crops. It does not harness the plant’s native genetic repertoire to create traits interest, rather it depends on the foreign source for gene of interest that mainly invites public concern. In the recent past, genome editing by engineered nucleases such as zinc finger nucleases (ZFNs) (Carroll, 2011), transcription activator like effector nucleases (TALENs) (Mahfouz et al., 2011; Li et al., 2012), and Clustered Regularly Interspaced Short Palindromic Repeats/CRISPR associated 9 (CRISPR/Cas9) (Cong et al., 2013) has proved a viable tool to alter the targeted site in the genome and has been widely used in several agricultural crops. CRISPR/Cas9-mediated improvement of horticultural crops has been discussed in the present review.

### Genome Editing by Sequence-Specific Nucleases

Genome editing technologies like ZFNs, TALENs, and CRISPR/Cas systems or RNA-guided engineered nucleases (RGEN) can be effectively employed to harness the plant’s native genomic repertoire by precise alteration in DNA sequences. While ZFNs and TALENs are limited by the technical complexity and limited efficiency, the CRISPR/Cas9 is simple with great efficiency. The CRISPR/Cas9 is a part of the adaptive immune system in prokaryotes which protects them against invading DNA such as viruses by cleaving the DNA in a sequence-dependent manner. The CRISPR/Cas9 system from *Streptococcus pyogenes* has been engineered to work with two components, a non-specific Cas9 endonuclease and a single guide RNA (sgRNA or gRNA) that directs Cas9 to specific genomic site depending on complementarity of protospacer with the target DNA resulting in double-strand breaks (DSBs) at target site (Jinek et al., 2012). These DSBs subsequently trigger cellular DNA repair pathways: error-prone non-homologous end joining (NHEJ) or homologous recombination (HR) pathway. Different site-specific DNA sequence modifications can be achieved depending on the repair pathway (Symington and Gautier, 2011). The repair by NHEJ mostly causes insertion or deletion (INDEL) mutation resulting in gene knockout if it is in the coding region. Alternatively, if DNA template having homology to the sequence surrounding the DSB is available, gene modification can be easily achieved through HR. The CRISPR/Cas9 system is easily programmable to target desired genomic site enabling DNA editing in the true sense. The necessary requirement for site-specific Cas9-catalyzed DNA cleavage in the genome is 20 nucleotides complementarity with gRNA sequence immediately followed by protospacer adjacent motif (PAM; Jinek et al., 2012). The major concern is the off target cleavage by Cas9 that can be avoided by selecting unique target site. CRISPR-Plant (Xie et al., 2014), GT-scan (O’Brien and Bailey, 2014), CRISPRdirect (Naito et al., 2015), and many more tools are available that help select the unique target site. Among them, CRISPRdirect is an ideal platform for horticultural crops providing specificity check for a large number of horticultural crops.

Owing to its high efficiency and simplicity, CRISPR/Cas9 is the potential tool for crop improvement and has been extensively used in different crops. To mention few in model plants, genes such as *AtPDS3*, *AtFLS2*, *AtADH*, *AtFT*, *AtSPL4*, *AtCHL1*, *AtTT4*, *AtAP1*, *AtGUUS*, and *AtBRI1* in *Arabidopsis thaliana*; *NbPDS*, *NbPCNA*, and *NbPDR6* in *Nicotiana benthamiana*; *OsSWEET11*, *OsSWEET14*, *OsCAO1*, *OsLAZY*, *OsROC5*, *OsSPP*, *OsYSA*, *OsPDS*, *OsBADH2*, *Oso2g23823*, *OsMPK2*, and *OsBEL* genes in *Oryza sativa* are efficiently targeted (Song et al., 2016). Although its applications are more visible in crops like rice and wheat, there are considerable examples in horticultural crops as well.

### CRISPR/Cas9 in Vegetable Crops

Most of the CRISPR/Cas9 experiments are done in tomato where 18 different genes have been targeted independently. The details of use of this technology is given in **Table 1**. The first CRISPR/Cas9-mediated genome editing in vegetable crops was reported in tomato in 2014 where *ARGONAUTE7* (*SlAGO7*) gene involved in leaf development was targeted (Brooks et al., 2014). *SlAGO7* gene was selected as a target for easy identification of edited plants as loss of function mutation of this gene results in a needle like or wiry leaves. Soon after this, developmental genes like SHORTROOT (SHR; root development; Ron et al., 2014); BLADE-ON-PETIOLE (SlBOP; inflorescence; Xu et al., 2016), and SELF PRUNING 5G (SP5G) and SELF PRUNING (SP; plant development; Soyk et al., 2017) were targeted for their functional validation in tomato. Interestingly, the tomato plant having both *sp5G* and *sp* genes mutated were very compact with early flowering. This experiment undoubtedly establishes the successful use of CRISPR/Cas9 in vegetable crop improvement. Developmental genes have also been targeted by CRISPR/Cas9 in other vegetables like *Brassica oleracea* (Lawrenson et al., 2015) and *Lactuca sativa* (Woo et al., 2015).

**Table 1:** Current status of genome editing in horticultural crops.

Serial No.	Plant	Target genes	Traits	Reference
(1)	*Solanum lycopersicum*	*ARGONAUTE7 (SlAGO7)*	Leaf development	Brooks et al., 2014
		*SHORT-ROOT (SHR)*	Root development	Ron et al., 2014
		*Ripening inhibitor (RIN)*	Fruit ripening	Ali et al., 2015b
		*Anthocyanin 1 (ANT1)*	Anthocyanin biosynthesis	Cermak et al., 2015
		*Phytoene desaturase (SlPDS), Phytochrome interacting factor (SlPIF4)*	Carotenoid biosynthesis	Pan et al., 2016
		*BLADE-ON-PETIOLE (SlBOP)*	Inflorescence	Xu et al., 2016
		*Self pruning 5G (sp5G), self pruning (sp)*	Plant development	Soyk et al., 2017
		*SlIAA9*	Parthenocarpy	Ueta et al., 2017
		*SlAGAMOUS-LIKE 6 (SlAGL6)*	Parthenocarpy	Klap et al., 2017
		*PHYTOENE SYNTHASE (PSY1)*	Fruit color	Hayut et al., 2017
		*MILDEW RESISTANT LOCUS o (Mlo)*	Powdery mildew resistance	Nekrasov et al., 2017
		*GABA-TP1*, *GABA-TP2*, *GABA-TP3*, *CAT9*, and *SSADH*	γ-Aminobutyric acid (GABA) synthesis	Li et al., 2017
(2)	*Solanum tuberosum*	*StMYB44*	Phosphate transport	Zhou et al., 2017
		*ACETOLACTATE SYNTHASE1 (StALS1)*	Herbicide resistance	Butler et al., 2016
		*Granule-bound starch synthase (GBSS)*	Starch quality	Andersson et al., 2017
(3)	*Brassica oleracea*	*Gibberellin3-beta-dioxygenase 1 (BolC.GA4.a)*	Plant development, fruit dehiscence	Lawrenson et al., 2015
(4)	*Lactuca sativa*	*BRASSINOSTEROID INSENSITIVE 2 (BIN2)*	Plant development	Woo et al., 2015
(5)	*Cucumis sativus*	*Eukaryotic translation initiation factor 4E (eIF4E)*	Virus resistance	Chandrasekaran et al., 2016
(6)	Grape	*Phytoene desaturase (VvPDS) gene*	Carotenoid biosynthesis	Nakajima et al., 2017
(7)	Citrus	*Phytoene desaturase (CsPDS)*	Carotenoid biosynthesis	Jia and Wang, 2014
		*CsLOB1 promoter*	Citrus canker resistance	Peng et al., 2017
		*CsLOB1*	Citrus canker resistance	Jia et al., 2017
(8)	Chrysanthemum	*CpYGFP*	Fluorescence	Kishi-Kaboshi et al., 2017
(9)	*Lotus japonicus*	*SYMRK*, *LjLb1*, *LjLb2*, *LjLb3*	Nitrogen fixation	Wang L. et al., 2016
(10)	Watermelon	*Phytoene desaturase (ClPDS)*	Carotenoid biosynthesis	Tian et al., 2017
(11)	*Salvia miltiorrhiza*	*diterpene synthase gene (SmCPS1)*	Tanshinone biosynthesis	Li et al., 2017


Genes of biosynthetic pathways in tomato such as *Anthocyanin 1* (*ANT1*) involved in anthocyanin biosynthesis (Cermak et al., 2015), *Phytoene desaturase* (*SlPDS*), *Phytochrome interacting factor* (*SlPIF4*) (Pan et al., 2016), and *Phytoene synthase* (*PSY1*) (Hayut et al., 2017) functioning in carotenoid biosynthesis were mutated by CRISPR/Cas9. The experiment by Hayut et al. (2017) demonstrated targeted recombination in heterozygote by crossing the two tomato plants stably expressing Cas9 and gRNA separately and also showed precise reshuffling of chromosomal segments between homologous chromosomes in somatic cells. This meticulously planned experiment opened a new door for CRISPR application where the inaccessible parts of a chromosome can be efficiently shuffled by targeted crossover to exploit the genes present in such locations.

Application of CRISPR/Cas9 for actual improvement of vegetable crops was reported very recently in 2017. Development of parthenocarpic tomato fruits with huge demand in processing industry was achieved simultaneously by two different groups. Klap et al. (2017) carried out knockout of *Slagamous-like 6* (*SlAGL6*) gene making mutant plants capable of producing parthenocarpic fruits under heat stress conditions that otherwise severely hamper fertilization-dependent fruit set. There was no other effect of mutagenesis making *SlAGL6* an attractive gene for facultative parthenocarpy. Alternatively, the other group has exploited a different approach by mutating *SlIAA9* gene involved in auxin signaling pathway that represses initiation of fruit development without fertilization (Ueta et al., 2017). This precise and rapid method of developing parthenocarpy can also be utilized in other horticultural crops like watermelon, pointed gourd, bitter gourd, etc. where seedless or less seeded fruits are in demand.

Clustered Regularly Interspaced Short Palindromic Repeats/CRISPR associated 9 is an important tool for developing resistance against various diseases. Plants harbor genes for disease resistance as well as genes conferring susceptibility to particular disease. A simple knockout of the susceptibility gene makes the plant resistant to specific disease. This has been achieved in tomato by knockout of *Mildew resistant locus o* (Sl*Mlo1*) gene conferring susceptibility to fungi *Oidium neolycopersici* causing powdery mildew disease (Nekrasov et al., 2017). Here, two positions of the gene were targeted by double sgRNA to create transgene-free powdery mildew-resistant tomato. Similar strategy has been earlier employed to develop resistance to powdery mildew in wheat (Wang et al., 2014). RNA viruses depend on the plant cell machinery to maintain their life cycle and eukaryotic translation initiation factor *eIF4E* is one of the major host factors required for virus multiplication. Silencing of *eIF4E* gene in tomato and melon has achieved broad spectrum RNA virus resistance earlier (Mazier et al., 2011; Rodríguez-Hernández et al., 2012). Following the similar strategy, broad spectrum resistance against viruses from potyviridae family was built in cucumber through knockout of *eIF4E* gene with the help of CRISPR/Cas9 using two gRNAs targeted at two different sites of *eIF4E* gene (Chandrasekaran et al., 2016). This strategy requires sufficient information about susceptibility genes and produces non-transgenic plants in contrast to plants where Cas9 and gRNA targeting to viral DNA are introduced in plants resulting in transgenic virus-resistant plants (Ali et al., 2015a). The second strategy of mimicking the CRISPR/Cas9 system in plants could be used to develop multiple virus resistance in by introducing single *Cas9* gene along with multiple gRNAs targeting each virus.

Clustered Regularly Interspaced Short Palindromic Repeats/CRISPR associated 9 has also been utilized for improving the quality of vegetables. Starch quality in potato is an important factor for food as well as many technical applications. The “waxy genotype” similar to that identified in maize producing only amylopectin containing starch (Klosgen et al., 1986) was developed in hexaploid potato by mutating *granule bound starch synthase* (*GBSS*) gene using CRSIPR/Cas9 (Andersson et al., 2017). The starch characterization showed that one of the genome-edited lines only produced amylopectin and completely lacks amylose confirming the knock-out of all the four alleles of *GBSS* gene. This experiment establishes the use of CRISPR/Cas9 for effective multi-allelic mutagenesis in polyploid crops. Similar multi-allelic mutagenesis has been achieved in potato by mutating *ACETOLACTATE SYNTHASE1* (*StALS1*) gene (Butler et al., 2016). It has been also exploited in potato for identification of *StMYB44* gene function making it an ideal tool for reverse genetics (Zhou et al., 2017).

Metabolic engineering in vegetables is an attractive field for improving their nutritional quality. But targeting multiple genes of a pathway for metabolic modifications is a challenging task. The recent report of manipulation of a four-carbon non-protein amino acid, γ-aminobutyric acid (GABA) shunt in tomato by simultaneously targeting five key genes significantly enhanced GABA accumulation in both the leaves and fruits (Li et al., 2017). Multiplexed CRISPR/Cas9 system was used here to obtain knockout lines of multiple genes in a single transformation experiment and thus establishes its application in metabolic engineering of vegetables.

### CRISPR/Cas9 in Fruit Crops

Although fruit crops are very important as a nutritional source, comparatively less attention has been given to their improvement because of their time-consuming breeding program owing to its perennial nature. Therefore, transgenics or genome engineering approaches are most suitable for them. Genome editing in fruit crops by CRISPR/Cas9 is an emerging field and very few reports have appeared so far (**Table 1**). It was reported in citrus by mutating *Phytoene desaturase* (*CsPDS*) gene of carotenoid biosynthesis pathway (Jia and Wang, 2014). The same group has further utilized CRISPR/Cas9 for developing citrus canker-resistant plants by knock-out of susceptibility gene *CsLOB1* (Jia et al., 2017). Different alleles of *CsLOB1* gene present in citrus contain the effector binding element (EBEPthA4), which is recognized by the main effector PthA4 of Xcc to activate *CsLOB1* expression. Therefore, another group adopted a different strategy to develop citrus canker resistance by targeting this effector binding element in promoter of *CsLOB1* (Peng et al., 2017). Other than citrus, genome editing by CRISPR/Cas9 has been demonstrated only in watermelon (Tian et al., 2017) and grape (Nakajima et al., 2017) by targeting *Phytoene desaturase* gene in both crops. Interestingly, the databases of target sites for CRSIPR/Cas9-based genome editing are being developed and one such grape-specific database “Grape-CRISPR” has been already published (Wang Y. et al., 2016). Such databases provide a great help and reduce background work for identifying target sites in a gene of interest. CRISPR/Cas9 applications in fruit crops are still at preliminary level but in view of the potential of this technology, many more reports showing applications in the improvement of fruit crops will float soon.

### CRISPR/Cas9 in Other Horticultural Crops

Genome editing by CRISPR/Cas9 is not limited to only fruits and vegetables but very recently it has been demonstrated in other horticultural crops like medicinal and ornamental plants. The first ornamental plant subjected to genome editing by CRISPR/Cas9 was a model legume *Lotus japonicus*. In this plant, symbiotic nitrogen fixation-related genes such as symbiosis receptor-like kinase and leghemoglobin loci (LjLb1, LjLb2, and LjLb3) were targeted and single as well as multiple gene-mutant plants were obtained, respectively (Wang L. et al., 2016). It was also reported that, for gene editing in nodules, the nodule-specific LjLb2 promoter is also effective in comparison to the constitutively active CaMV35S promoter. Another example is of a very important hexaploid ornamental plant *Chrysanthemum morifolium* (chrysanthemum). The transgenic chrysanthemum stably expressing *yellowish-green fluorescent protein* (*CpYGFP*) gene was utilized for genome editing where *CpYGFP* gene was mutated by two sgRNAs targeted at different positions (Kishi-Kaboshi et al., 2017). They have also suggested that PcUbi promoter for Cas9 and AtU6 promoter for sgRNA are suitable for carrying out genome editing in chrysanthemum. Chrysanthemum is also a natural source of insecticides and successful genome editing in this plant has paved the way for manipulation of biosynthetic pathways leading to new, more efficient, and broad spectrum insecticides. Besides these ornamental plants, the Chinese medicinal plant *Salvia miltiorrhiza* has been edited through CRISPR/Cas9 to knock-out diterpene synthase gene (*SmCPS1*) involved in tanshinone biosynthesis and successfully developed plants lacking tanshinones without affecting other phenolics metabolites (Li et al., 2017). Genome editing in medicinal plants will further help in studying the biosynthetic pathways of secondary metabolites and improvement of their yield and quality.

### Future of Genome Editing in Horticultural Crops

Improvement of horticultural crops is focused on some of the important aspects like an increase in yield, resistance to pest and diseases, tolerance to various abiotic stresses, enhanced shelf life, processing quality, esthetic value, enhanced nutrition value, etc. Some of these traits are monogenic while others are polygenic and complex that are difficult to improve and also time consuming by traditional breeding. With CRISPR/Cas9, it is possible to mutate multiple genes in plant genome and at the same time it is also possible to insert multiple genes through HR. CRISPR/Cas9-mediated virus resistance has been developed in the plant by transferring Cas9 gene and a sgRNA specific for viral DNA (Ali et al., 2015a). Similarly, broad spectrum virus resistance can be developed in plants by inserting the multiple sgRNAs targeting genomic regions of all the viruses. This strategy of developing virus resistance will be a boon to many horticultural crops where the source of virus resistance is not present in available germplasm. Moreover, one can think of even developing immunity in plants against viruses by transferring whole CRISPR system of prokaryote including all other genes required for recognizing foreign DNA and inserting them in CRISPR locus. This will solve the problem of frequent virus resistance breakdown in plants due to continuous evolution in viral genomes. Eliminating the expression of susceptibility gene is one of the ways to develop disease resistance, but sequence information of the susceptibility gene is the prerequisite here. In crops where sequence information about both resistance as well as susceptibility gene is available, one can achieve simultaneous deletion of susceptibility gene and insertion of resistance gene at deletion site using this tool. Further, multiple gene transfer through HR paves the way for inserting the whole biosynthetic pathways into model plants for synthesizing useful phytochemicals. Thus, metabolic engineering can be easily achieved by CRISPR/Cas9 to improve vegetables, fruits, and medicinal plants which are very important source of nutrition and health promoting factors. Biosynthesis of important phenolic compounds like anthocyanins can be enhanced by modifying some of the genes involved in its synthesis (Xiong et al., 2015). Many desired traits can also be engineered in horticultural crops. In peas, as the seed matures it loses the sweetness due to the conversion of sugar to starch which can be inhibited by mutating the *starch branching enzyme1* (*SBE1*) gene (Bhattacharyya et al., 1990) to develop something like “stay sweet” trait in peas. In cucumber, breeders aim to develop varieties lacking cucurbitacin that causes bitterness. Knock-down of the gene/s responsible for cucurbitacin synthesis (Shang et al., 2014) can develop cucumbers lacking bitterness. Similarly, the anti-nutritional factors present in crops like vegetable soybean and leafy vegetables can be removed while nutritional factors can be enhanced by manipulating their biosynthetic pathways. Browning due to oxidation of phenolic compounds by polyphenol oxidase (PPO) in fruits and vegetables results in negative effects on color, taste, flavor, and nutritional value. This problem can be tackled by mutating the *PPO* gene as done in non-browning mushroom. These are only a few potential applications and there could be several other crop-specific applications.

Availability of whole genome and transcriptome sequences in several horticultural crops is definitely a boosting factor for application of such advanced tools where sequence information is a prerequisite. The higher efficiency of targeted mutagenesis with CRISPR/Cas9 tool will help to identify gene functions in such crops that can further help to design better genome editing strategies. Sufficient sequence information is still not available in a large number of horticultural crops which is the major challenge and limiting factor in the CRISPR/Cas9 applications. Transformation and regeneration protocols are also a basic requirement for genome editing work. This technique has been standardized in many crops (Cardi et al., 2017) while there are a large number of horticultural crops where standard protocols are not available making it another limiting factor. With advancements in tissue culture research, standardization of regeneration protocols in all the crops will certainly gain the speed in near future to reap the benefits of this wonderful technology. Genome editing is more or less similar to mutation breeding but in a very precise manner and thus it is entirely different from transgenesis. Although genetically edited mushroom is approved commercially in the United States, public acceptance in European countries and countries like India is a challenging issue. Moreover, there are no specific guidelines available for regulation of genetically edited crops. Despite all these challenges, in the long run, CRISPR/Cas9 will undoubtedly bring revolution in the breeding of horticultural and other agricultural crops.

## Author Contributions

SK and BS conceived the idea. SK and OG wrote the manuscript. SK, AS, and PS critically evaluated the manuscript. All authors approved the manuscript.

## Conflict of Interest Statement

The authors declare that the research was conducted in the absence of any commercial or financial relationships that could be construed as a potential conflict of interest.
